# Supplementary material for: The Role of Substrate on Thermal Evolution of Ag/TiO2 Nanogranular Thin Films
Source: Nanomaterials (Basel). 2021 Aug 31;11(9):2253. doi: 10.3390/nano11092253 (PMC8471301; doi:10.3390/nano11092253)
Supplement: Supplementary file 1 [file nanomaterials-11-02253-s001.zip › nanomaterials-1314065-supplementary.pdf]

The Role of Substrate on Thermal Evolution of Ag/TiO_2 Nanogranular Thin Films

Vincenzo Balzano, Emanuele Cavaliere, Mattia Fanetti, Sandra Gardonio, Luca Gavioli

[1] Interdisciplinary Laboratories for Advanced Materials Physics (i-LAMP), Dipartimento di Matematica e Fisica, Università Cattolica del Sacro Cuore, Via Musei 41, 25121 Brescia, Italy; vincenzo.balzano@unicatt.it (V.B.); emanuele.cavaliere@unicatt.it (E.C.)
[2] Materials Research Laboratory, University of Nova Gorica, Vipavska 11c, 5270 Ajdovšˇcina, Slovenia; mattia.fanetti@ung.si (M.F.); sandra.gardonio@ung.si (S.G.)
* Correspondence: luca.gavioli@unicatt.it

Electronic Supplementary Information

1) Characterization, annealing and evolution of the films

Here we show a schematic diagram of the annealing treatments, characterization steps and main properties of the Ag/TiO_2 samples deposited on silica and sapphire. **Fig. S1-a** shows the main characterization techniques employed: Raman spectroscopy, Spectrophotometry and AFM (only on SiO_2) were performed after every annealing step. The samples were annealed for 1h at temperatures ranging from 100 to 900°C, with ΔT = 100°C between each step. SEM, EDX, XPS and AFM were performed on both samples at the end of the last annealing step (900°C) in order to investigate the different properties of the samples depending on their deposition substrates. **Fig. S1-b** summarizes the main differences in behaviour between the samples, arising from the heat treatment at 600°C and higher.

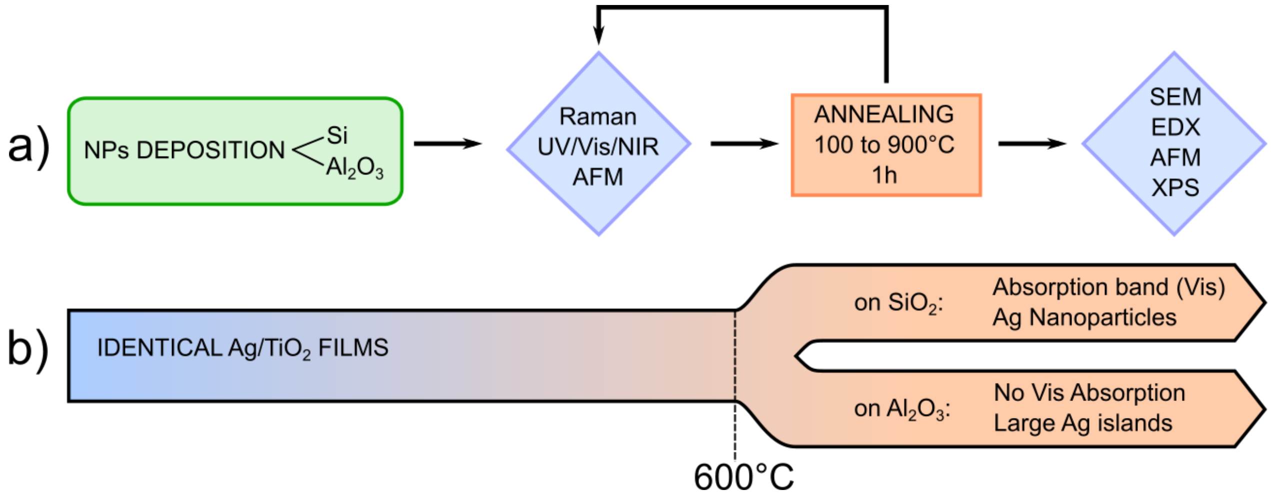

Fig. S1 a) Schematic diagram of the deposition and annealing of the Ag/TiO_2 films on silica and sapphire and of the characterization techniques employed in the study. **b)** Brief overview of the different temperature-induced optical and morphological evolution of the Ag/TiO_2 films on silica and sapphire.

2) Size distribution of the Ag/TiO_2 NPs synthesized by SCBD

Here we report an analysis of the dimensions of the single Ag/TiO_2 nanoparticles produced by the SCBD process, which are the starting point for the deposition of the Ag/TiO_2 nanogranular films analysed in this work. **Fig. S2a** shows the normalized size distribution of the NPs on the vertical (z) axis of the sample, while **Fig. S2b** shows the same distribution calculated on the lateral (x-y) direction. Both distributions were obtained via AFM on a dedicated sample composed of sparse NPs deposited on a silicon wafer. Before deposition, the silicon wafer was treated with a collodion solution in order to remove contaminations and allow us a precise measure of the background roughness. The density of the deposited NPs was 160 ± 12 NPs/μm^2. The AFM analysis of the particle size was performed over a set of 10 512 x 512 px^2 images with a scan area of 2 x 2 μm^2. A Z-threshold mask was used in order to mark the NPs on the surface of the sample. The NP size distribution on the z axis is multimodal, presenting three main components: each component follows a lognormal distribution, as expected from this type of deposition technique [1]. The first mode is composed by NPs with a mode radius of 0.42 ± 0.01 nm. Their small dimension makes them hardly distinguishable from the background noise, meaning that the total number of NPs in this mode is likely to be an overestimation. The mode radius of the NPs in this distribution is compatible with previous measurements of TiO_2 NPs produced by SCBD. The second distribution has a mode radius of 1.49 nm, while the third distribution has a mode radius of 2.81 nm. The NPs in the third mode represent the vast majority

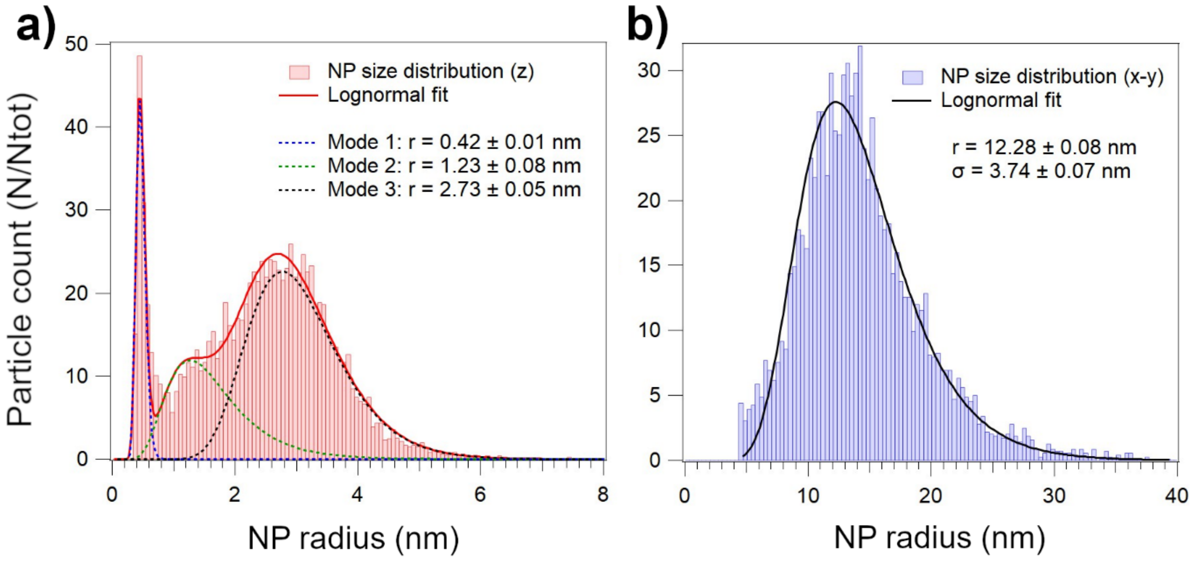

Fig. S2 a) Normalized size distribution of Ag/TiO_2 NPs on Si wafer calculated on the z axis of the film. **b)** Normalized size distribution of Ag/TiO_2 NPs on Si wafer calculated along the x-y direction of the film

(> 94%) of the precursor cluster in volume. The radius of the NPs in this last mode is compatible with previous literature regarding 50-50 Ag/TiO_2 nanoparticles produced by SCBD, where the mean radius of the Ag NPs measured by HAADF-STEM was 0.75 nm. Since the Ag NPs in this material are partially of completely enclosed in TiO_2, as observed in TEM images, it is reasonable that the mode radius of the Ag-TiO_2 nanoparticles is higher than that of Ag only.

3) **XPS analysis**

Fig. S3a shows an XPS survey of the Ag/TiO_2 thin films as deposited (green) and after the last annealing step at 900°C for 1h (red and black curves). At deposition, the XPS spectra of the film deposited on silica is equivalent to previous spectra obtained on the same material on a sapphire substrate [2], as expected by films deposited by SCBD. **Fig. S3b** shows the O1s core level peaks for both silica and sapphire. Alongside the component related to TiO_2, the silica-deposited film annealed at 900°C for 1h also show a further peak due to the SiO_2 bonding. This indicates a partial exposure of the silica substrate through the cracks formed in the film and observed in the AFM data, induced by the grain coarsening. **Fig. S3c** shows the TiO_2 core level of the as-deposited and annealed films. The binding energies of the as-deposited sample is compatible with previous measurements on the same material [2]. After annealing, the TiO_2 peak is shifted by 0.4 eV towards lower binding energies. This shift could be attributed to the formation of anatase and rutile crystalline phases observed by Raman spectroscopy for annealing temperatures of 700°C and higher.

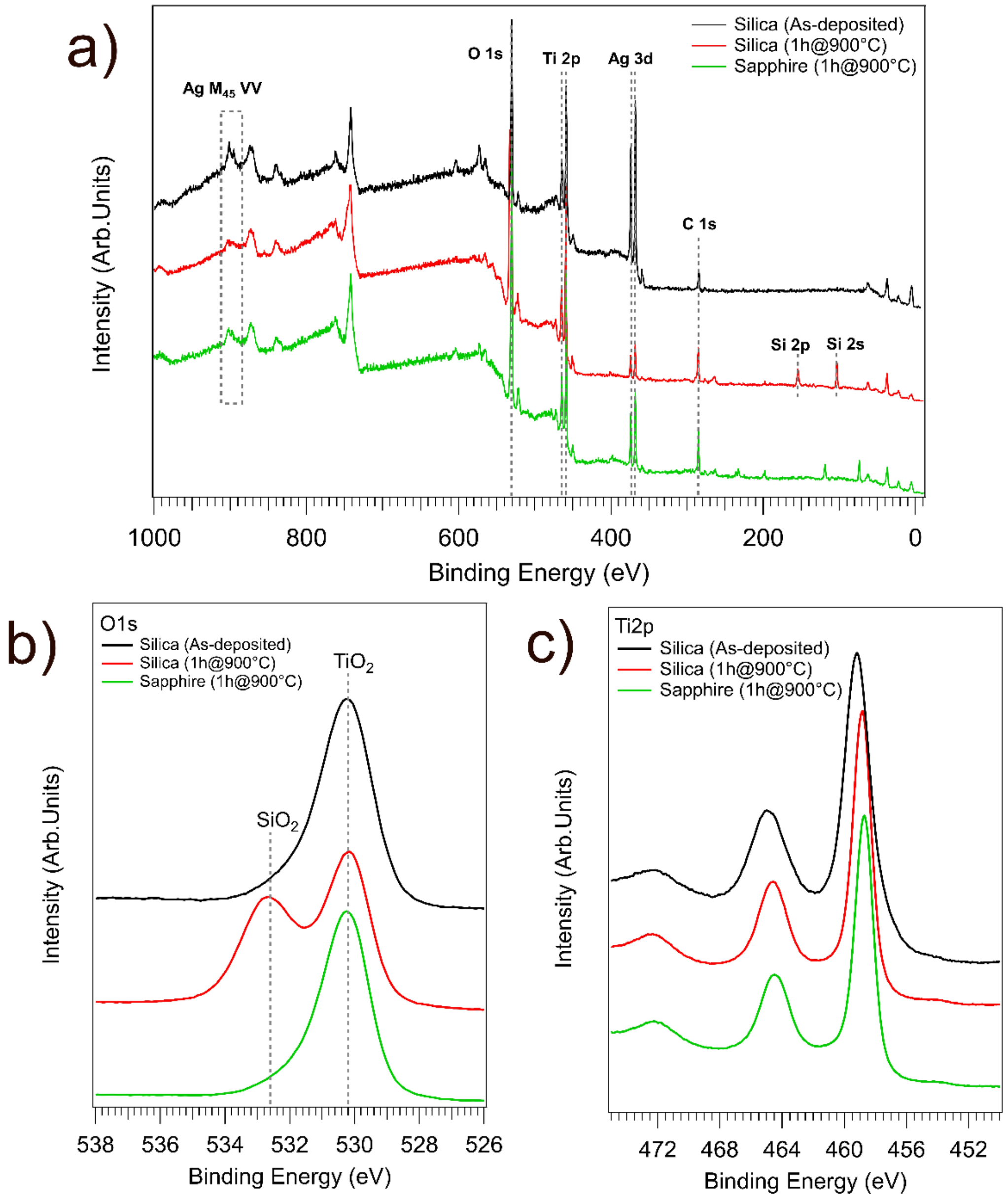

Fig. S3. XPS analysis of the 50nm thick Ag/TiO_2 films on sapphire and silica after annealing at 900°C for 1h compared with an as-deposited film on silica: **a)** XPS survey of the samples; **b)** Analysis of the O1s peaks (The reference binding energies for TiO_2 and SiO_2 are reported in a dotted line), and **c)** Analysis of the Ti2p peaks.

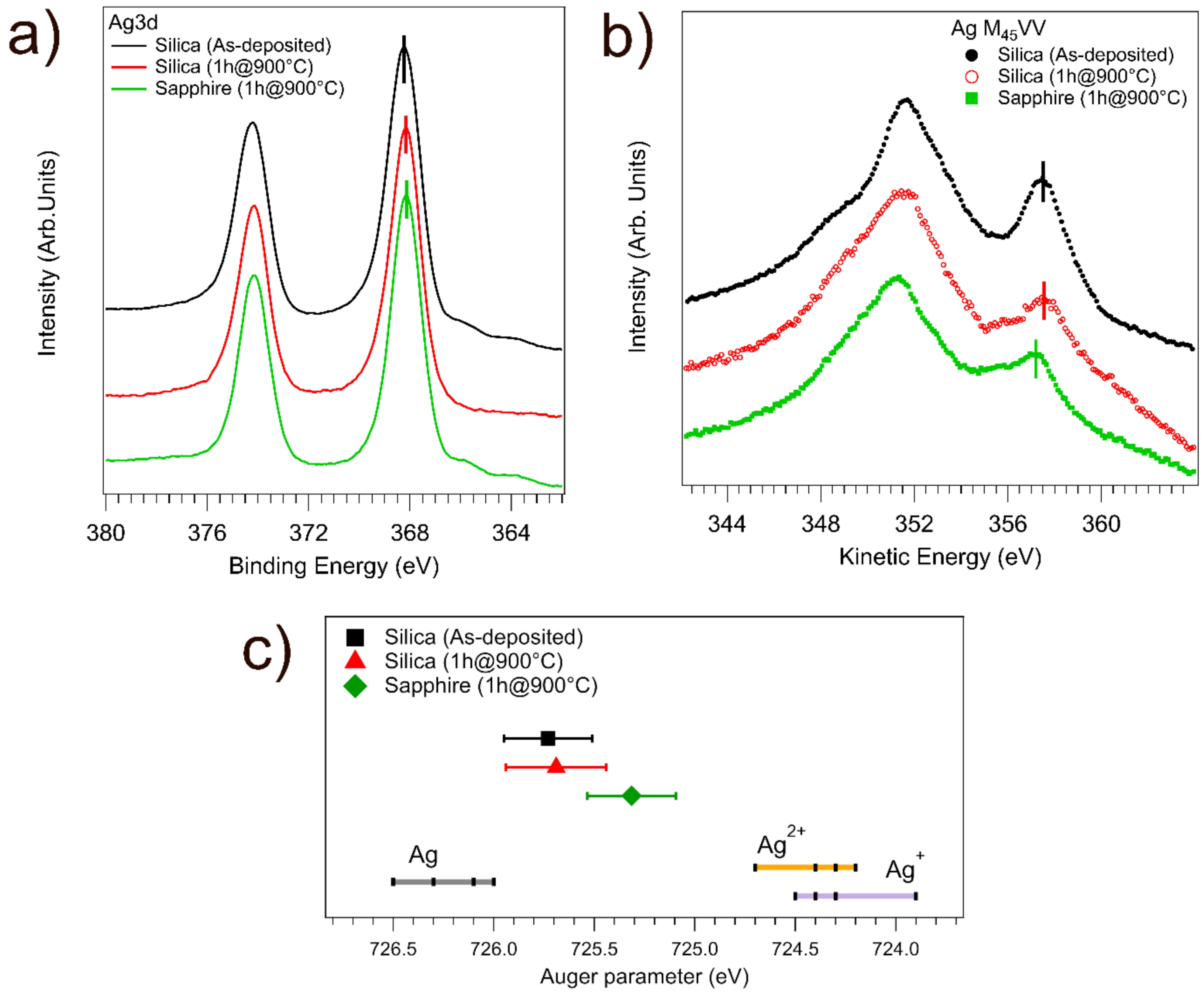

Fig. S4. a) XPS analysis of the Ag3d peak for the Ag/TiO_2 samples on silica and sapphire after annealing at 900°C for 1h compared to an as-deposited film on silica. The spectra are normalized for comparison. **b)** Auger MNN emission line for the samples. **c)** Calculated Auger parameters of the samples compared to the literature.

Fig. S4a shows the spectra of the Ag3d core levels of the samples, while **Fig. S4b** shows the corresponding Auger $M_{45}VV$ transition lines. The oxidation state of Ag was evaluated by calculating the Auger parameters. **Fig. S4c** shows the obtained experimental Auger parameters for this work compared with the literature [3–6]. The calculated Auger parameter for the as-deposited film is 725.73 ± 0.21, which is compatible with previous results on the same material [2], highlighting the metallic nature of the Ag NPs at deposition. For the silica-deposited sample, after the last annealing step at 900°C the Auger parameter is 725.7 ± 0.25, which is basically unchanged with respect to the RT film, suggesting that Ag is not oxidizing. Conversely, on the sapphire-deposited sample, the Auger parameter is 725.32 ± 0.22, which is suggesting a partial oxidation of the Ag particles. This can be explained by recalling that annealing at 900°C for 1h in this sample results in the formation of 570 nm-wide Ag clusters on top of film surface, which are more exposed to air compared to the scattered NPs observed in the silica-deposited film, thus leading to a higher oxidation of Ag.

4) **Ag clusters on sapphire**

Fig. S5 shows an AFM image of the Ag/TiO_2 thin film on sapphire after annealing at 900°C for 1h, while **Fig. S6** shows a SEM image taken on the same film. The bright objects in both images represent Ag aggregates which are formed during the annealing process. In order to calculate the mean height of the clusters, we applied a z-threshold mask to the AFM image to separate the clusters from the background and then we obtained the maximum z height of every grain over the mean level of the background. The mean height of the clusters thus obtained was 360 ± 8 nm. The lateral dimension of the clusters was calculated in a similar way using SEM data: in this case, the high contrast between the clusters and the background was used to obtain a precise measurement of their projected area. The calculated mean diameter for the Ag clusters was 570 ± 12 nm.

5) **Complete Raman spectra of Ag/TiO_2 thin films on silica and sapphire**

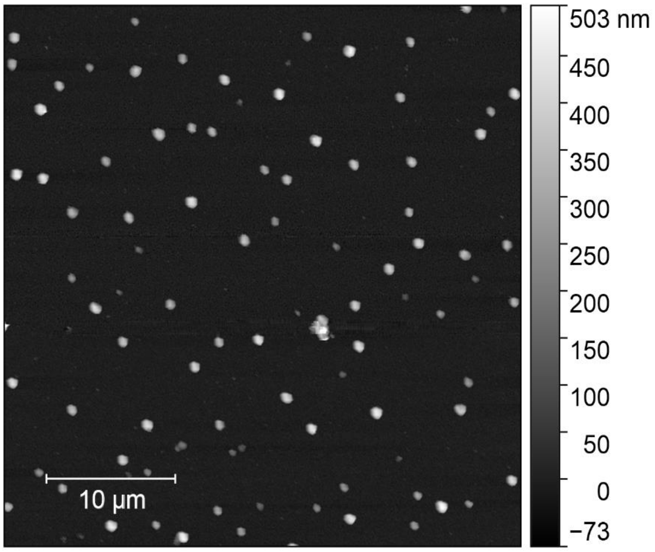

Fig. S5. AFM image of Ag clusters on the surface of the sapphire-deposited Ag/TiO_2 thin film after annealing at 900°C for 1h.

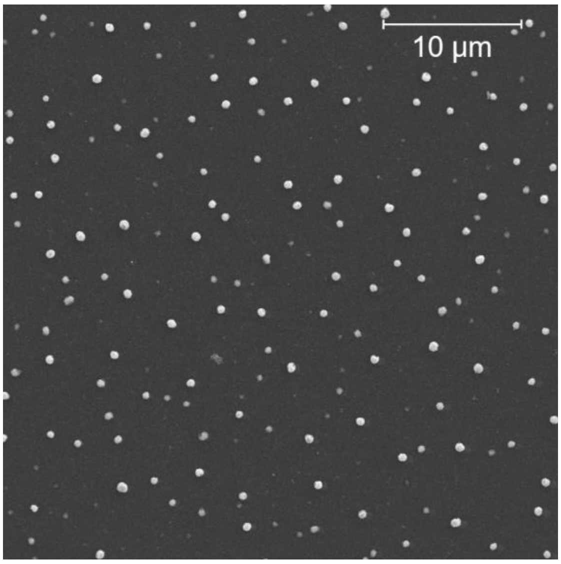

Fig. S6. SEM image of Ag clusters on the surface of the sapphire-deposited Ag/TiO_2 thin film after annealing at 900°C for 1h.

Fig. S7 and **S8** show the Raman spectra of the Ag/TiO_2 film on silica and sapphire, respectively, as a function of the annealing temperature. At room temperature, both films are amorphous as testified by the absence of anatase or rutile peaks. In the sapphire-deposited films, the single peak located at 400 cm^{-1} and the double peak at 1400 cm^{-1} are due to the sapphire substrate. The large Raman band at 750 cm^{-1} is not compatible with any of the Ag or Ti oxides and could be attributed to surface contamination. Also, this band disappears after annealing at 300°C. Both films remain amorphous for annealing temperatures up to 600°C. Above 700°C both samples show presence of both anatase and rutile phases. Moreover, a complex structure can be observed at wavelengths 700 to 900 cm^{-1} on both samples, which is not related to the formation of anatase or rutile. The shape and position of this structure changes significantly after every annealing step, especially on the silica-deposited sample, which makes it difficult to identify its nature. Mosquera et al. [7] have observed a similar structure in Ag/TiO_2 with an Ag/(Ag+Ti) atomic ratio of 40% annealed at 600°C and higher: according to the authors, this structure is originated by the silicon substrate. This, however, cannot explain why we also see them on the sapphire-deposited film.

References

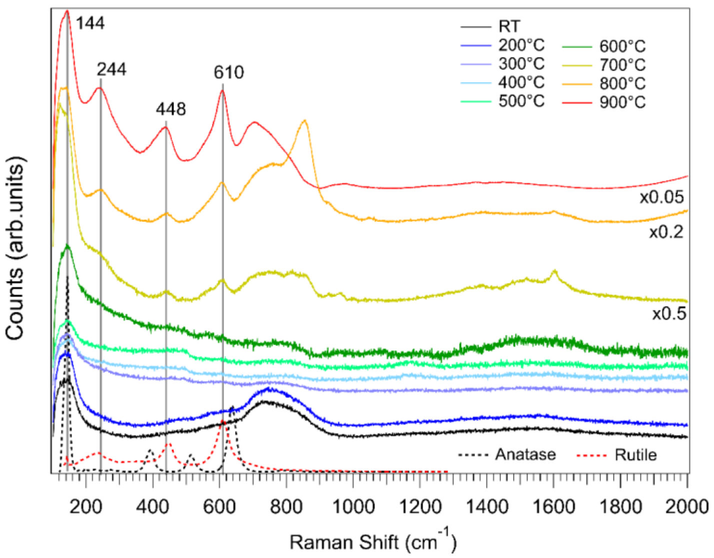

Fig. S7. Raman spectra of the Ag/TiO_2 thin film on silica as a function of the annealing temperature. The spectra of bulk anatase and rutile are shown as reference.

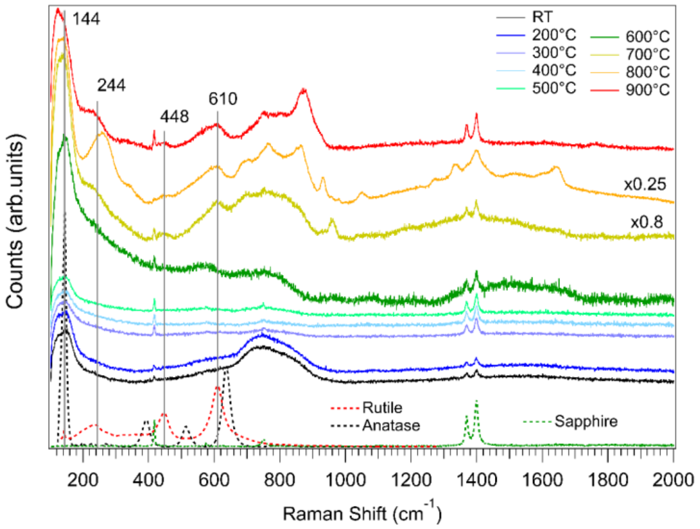

Fig. S8. Raman spectra of the Ag/TiO_2 thin film on sapphire as a function of the annealing temperature. The spectra of bulk anatase, rutile and single-crystal sapphire are shown as reference.

1. Piseri, P.; Podestà, A.; Barborini, E.; Milani, P. Production and Characterization of Highly Intense and Collimated Cluster Beams by Inertial Focusing in Supersonic Expansions. *Review of Scientific Instruments* **2001**, *72*, 2261–2267, doi:10.1063/1.1361082.

2. Benetti, G.; Cavaliere, E.; Canteri, A.; Landini, G.; Rossolini, G.M.; Pallecchi, L.; Chiodi, M.; Van Bael, M.J.; Winckelmans, N.; Bals, S.; et al. Direct Synthesis of Antimicrobial Coatings Based on Tailored Bi-Elemental Nanoparticles. *APL Materials* **2017**, *5*, 036105, doi:10.1063/1.4978772.
3. Waterhouse, G.I.N.; Bowmaker, G.A.; Metson, J.B. Interaction of a Polycrystalline Silver Powder with Ozone. *Surf. Interface Anal.* **2002**, *33*, 401–409, doi:10.1002/sia.1223.
4. Ferraria, A.M.; Carapeto, A.P.; Botelho do Rego, A.M. X-Ray Photoelectron Spectroscopy: Silver Salts Revisited. *Vacuum* **2012**, *86*, 1988–1991, doi:10.1016/j.vacuum.2012.05.031.
5. Powell, C.J. Recommended Auger Parameters for 42 Elemental Solids. *Journal of Electron Spectroscopy and Related Phenomena* **2012**, *185*, 1–3, doi:10.1016/j.elspec.2011.12.001.
6. Kaushik, V.K. XPS Core Level Spectra and Auger Parameters for Some Silver Compounds. *Journal of Electron Spectroscopy and Related Phenomena* **1991**, *56*, 273–277, doi:10.1016/0368-2048(91)85008-H.
7. Mosquera, A.A.; Albella, J.M.; Navarro, V.; Bhattacharyya, D.; Endrino, J.L. Effect of Silver on the Phase Transition and Wettability of Titanium Oxide Films. *Sci Rep* **2016**, *6*, 32171, doi:10.1038/srep32171.
